# The impact of third-party authorization requirements on abortion-related outcomes: a synthesis of legal and health evidence

**DOI:** 10.1186/s12889-023-16307-1

**Published:** 2023-10-23

**Authors:** Fiona de Londras, Amanda Cleeve, Maria I. Rodriguez, Alana Farrell, Magdalena Furgalska, Antonella F. Lavelanet

**Affiliations:** 1 Birmingham Law School, University of Birmingham, B15 2TT Birmingham, UK; 2 Women’s and Children’s Health, Karolinska Institute, Stockholm, Sweden; 3 South General Hospital (Södersjukhuset), Stockholm, Sweden; 4 Department of Obstetrics and Gynecology, Oregon Health and Science University, Portland, OR USA; 5 York Law School, University of York, York, UK; 6 Department of Sexual and Reproductive Health and Research, UNDP-UNFPA-UNICEF-WHO-World Bank Special Programme of Research, Development and Research Training in Human Reproduction (HRP), World Health Organization, Geneva, Switzerland

**Keywords:** Abortion, Reproductive rights, Third party authorization requirements, Abortion law

## Abstract

**Objectives:**

This review synthesizes legal and health evidence to demonstrate the health and human rights impacts of third-party authorization requirements (TPAs) on abortion seekers.

**Results:**

The synthesized evidence substantiates the pre-existing position in international human rights law that requirements that abortion be authorized by third parties like parents, spouses, committees, and courts create barriers to abortion, should not be introduced at all, or should be repealed where they exist.

**Conclusions:**

The review establishes that rights-based regulation of abortion should not impose TPAs in any circumstances. Instead, the provision and management of abortion should be treated in a manner cognizant with the general principles of informed consent in international human rights law, presuming capacity in all adults regardless of marital status and treatment sought, and recognizing the evolving capacity of young people in line with their internationally-protected rights.

**Supplementary Information:**

The online version contains supplementary material available at 10.1186/s12889-023-16307-1.

## Background

It is well established in both medical ethics and in international human rights law that nobody may be subjected to any health intervention unless they consent to it, and that such consent must be freely given and fully informed [1, para 8; 2–5; 73]. As the UN Special Rapporteur on the right of everyone to the enjoyment of the highest attainable standard of physical and mental health has put it [6], “Guaranteeing informed consent is fundamental to achieving the enjoyment of the right to health through practices, policies and research that are respectful of autonomy, self-determination and human dignity”. Rooted not only in autonomy and agency, the general principle of consent to healthcare interventions is “an integral part of respecting, protecting and fulfilling the enjoyment of the right to health” [6, para 18]. It not only prevents non-consensual interventions but is also a mode of enacting the right to privacy and confidentiality on health matters, which is fundamental to ensuring that all people can seek and avail of healthcare without apprehension.

Mental capacity is generally presumed in adults. The general principle of informed consent is applied in a modified way to persons who are considered to have reduced mental capacity (also known in some settings as competency) such as minors or persons with disabilities or health conditions that impact on their perceived ability to make autonomous decisions as to health interventions, all of whom are guaranteed the right to non-discrimination in health care [1, paras 18, 26]. Mental capacity is usually determined by reference to the person’s ability to understand, retain, believe, and weigh up information provided to them in the process of deciding about whether to proceed with an offered intervention or course of action [6, para 10]—in the case of abortion, whether to continue with pregnancy or to end their pregnancy through induced abortion—or other cognitive abilities. For persons with reduced mental capacity, medical ethics and human rights law generally provide that they should be supported to understand and be full participants in decisions about their health care [7, 8, para 41; 9]. Only in very exceptional cases—where the person has no mental capacity or in situations of emergency—might a third party’s judgement substitute that of the person whose treatment is in question.

These general principles of consent to health and medical interventions have long been under strain in sexual and reproductive healthcare including abortion. Lavelanet et al. [10]. found that 105 countries of 158 analyzed required authorization by one or more health worker for abortion to be lawfully provided, while one third of countries that permit abortion required parental authorization for minors, and twelve required spousal consent. Such third-party authorization requirements are found in jurisdictions all over the world, and within abortion laws that are broadly considered liberal or permissive as well as those considered generally restrictive. In practical terms this means that a spouse, a parent, a court, a committee, a police officer, a medical professional, or another specified authority can effectively override one’s stated preference to end pregnancy through abortion by refusing to ‘authorize’ it. These arrangements are known as third party authorization requirements (TPAs) and run counter to the principle that—absent a lack of mental capacity—it is the ‘patient’ alone who decides whether to undergo an intervention or treatment.

International human rights law bodies have concluded that requirements for parents, spouses, committees, and courts to authorize abortion create barriers should not be introduced at all, or, where they do exist, should be repealed. As a matter of international human rights law, states may not restrict women’s access to health services on the ground that they do not have the authorization of husbands, partners, parents or guardians, or health authorities, because they are unmarried, or because they are women [11, paras 14, 21; 2 paras 41, 43; 3]. Furthermore, states must recognize children’s and adolescents’ evolving capacity and their associated ability to take decisions that affect their lives [12, Article 5].

This review aims to address gaps in existing knowledge about the health and non-health outcomes that relate to TPAs. Rather than doing this by means of a classic systematic review, we have synthesized evidence from existing studies (i.e., data extracted from included studies) and international human rights law (i.e., standards articulated in and by international human rights law sources and bodies) according to a previously published methodology that was developed for this purpose [13] and which is appropriate for complex interventions with multiple effects, including non-linear and context-dependent effects [14]. Interventions of this kind often interact with one another, meaning that outcomes related to one individual or community may be interdependent, and could be positively or negatively impacted by the presence, arrangement, and implementation of institutions, resources, and people within the broader system in which they operate [15]. This review is one of seven reviews that was conducted as part of the evidence base for the WHO’s *Abortion Care Guideline* [16].

Consistent with the approach in the *Abortion Care Guideline* [16], we use the terms women, girls, pregnant women [and girls], pregnant people, and people interchangeably in this review to include all those with the capacity to become pregnant.

## Methods

### Identification of studies and data extraction

This review examined the impact of the TPAs on pregnant people seeking abortion. Having undertaken a preliminary review of the literature [17], scholars and experts from law, policy, and human rights codeveloped a search strategy and outcomes of interest. Our outcomes of interest were delayed abortion, continuation of pregnancy, opportunity costs, unlawful abortion, self-managed abortion, anticipated family disharmony, anticipated exposure to violence or exploitation, anticipation reproductive coercion, and system costs.

Using a combination of MeSH terms and keywords, we searched English language texts in PubMed, HeinOnline, JStor, and the search engine Google Scholar. As the second edition of the WHO’s *Safe Abortion Guidance* included data up until 2010, we limited our search to papers published in English from 31 to 2010 to 2 December 2019. An updated search of the same databases was undertaken through July 2021. All studies that included original data collection or analysis could be included. Thus, we included quantitative studies, qualitative and mixed-methods studies, reports, PhD theses, and economic or legal analyses, both comparative and non-comparative. Masters’ theses and abstracts were excluded.

The full review team was made up of 6 members (MF, AF, FdL, AC, MR and AL). AL and FdL developed the PICO. Two reviewers (MF and AF) conducted an initial screening of the literature. Titles and abstracts were first screened for eligibility using the Covidence® tool following which MF and AF reviewed full texts. Full texts were then reviewed. FdL confirmed that these manuscripts met inclusion criteria. FdL and AC extracted data. Any discrepancies were reviewed and discussed with AL and MR. Where they arose, discrepancies were resolved through consensus.

In accordance with our previously published methodology for the effective integration of human rights as evidence in systematic reviews for guideline development, [13] we reviewed international human rights law to identify relevant human rights standards. These were standards that referred expressly to TPAs for sexual and reproductive healthcare including abortion, and standards that outlined states’ relevant general obligations as they relate to sexual and reproductive healthcare. As we have described elsewhere [13], we identified these standards through analysis of treaties, general comments, opinions of treaty monitoring bodies, and reports of special procedures.

We then integrated the evidence from the studies and international human rights law to identify the implications of TPAs in abortion law and policy. This allowed us to develop a full understanding of (a) which human rights standards are engaged by TPAs, (b) whether the included studies suggest that TPAs have positive or negative effects on the enjoyment of those rights, and (c) where no data is identified from the manuscripts against outcomes of interest, whether human rights law provides evidence that can further elucidate the impacts and effects of TPAs.

### Patient and Public Involvement

It was not appropriate or possible to involve patients or the public in the design, or conduct, or reporting, or dissemination plans of our research.

### Types of third-party authorizations

As already mentioned, a wide range of TPA requirements are found in abortion law and policy. This review considers five such requirements: parental involvement, parental notification, parental ‘consent’, judicial bypass, and ‘spousal consent’ requirements. These types of authorization were identified deductively from the results of the first search for evidence undertaken for this review, and reflect the kinds of authorizations found in the manuscripts identified through that search strategy. Although specifically sought in the manuscript search, studies containing original data on the impacts of other forms of TPA (such as health worker authorization or general requirements for judicial authorization to access abortion) were not identified.

We use the term ‘parental involvement’ to identify interventions the precise nature of which was not specified in the studies, but which comprised formal requirements for parental authorization (known in much of the US-based literature as ‘parental consent’) and/or ‘mere’ notification requirements. ‘Parental involvement’ requirements are broadly considered within the definition of TPA for the purposes of this analysis because they constitute the legally mandated involvement of a third party who has the legal and/or relational authority to (seek to or actually) determine the pregnant person’s effective access to lawfully available abortion. ‘Parental notification’ requirements mandate that a parent or guardian be made aware that a minor is seeking abortion, although they do not provide that a parent’s authorization must be secured before abortion can be provided. For example, the Colorado Parental Notification Act (2003) requires healthcare providers to provide a parent or guardian with at least 48 hours written notification of a young person’s scheduled abortion. ‘Parental consent’ requirements do mandate that a parent’s or guardian’s authorization is required for a lawful abortion to be provided. Importantly, these requirements are separate to any general rules that may apply to a minor’s capacity to (refuse) consent to healthcare interventions. They are particular to the context of abortion and apply simply on the basis that the person seeking abortion is under a specified age (usually 18), without any reference to their mental capacity to consent to abortion as a healthcare intervention. In the state of Kansas, USA, for example, the law provides that a minor may not receive abortion care without the prior, “notarized written consent of the minor and both parents or the legal guardian of the minor” (KSA 65-6705). ‘Judicial bypass’ is the term conventionally used to describe a process that allows a minor to ‘bypass’ a legal requirement for parental authorization of abortion by substituting it with judicial authorization; this mechanism is typically but not exclusively found in the law of some states in the United States of America. While minors can use judicial bypass to avoid informing their parent or guardian about their pregnancy and desire to access abortion, judicial bypass is itself a form of TPA, as access to abortion is conditional on authorization from a court (i.e., a third party). Finally, ‘spousal consent’ requirements mandate that a woman cannot access abortion unless her husband authorizes it (or, in the commonly used term, ‘consents’ to it). Such requirements apply regardless of the pregnant person’s mental capacity to consent to healthcare interventions. In Japan, for example, the Maternal Health Act 1996 permits abortion in certain circumstances and with the consent of the pregnant person’s spouse.

### Analysis

We organized data from the included studies by reference to our study outcomes and presented this in evidence tables. These tables presented the association of each study on the outcome together with an overall conclusion from the data relevant to the outcome of interest. We then applied both general human rights standards and those specifically relating to TPAs to these outcomes by assessing whether the evidence from the included studies indicated that TPAs had effects that were incompatible with established requirements of international human rights law. To summarize the effect of the intervention, across all study designs, we used and applied a visual representation of effect direction. The direction of the evidence was illustrated by a symbol which indicated whether, in relation to that particular outcome, the evidence extracted from a study suggested an increase (▲), decrease (⊽), or no change in the outcome (○). The symbol did not indicate the magnitude of the effect [13, 15].

## Results

**Fig. 1:**
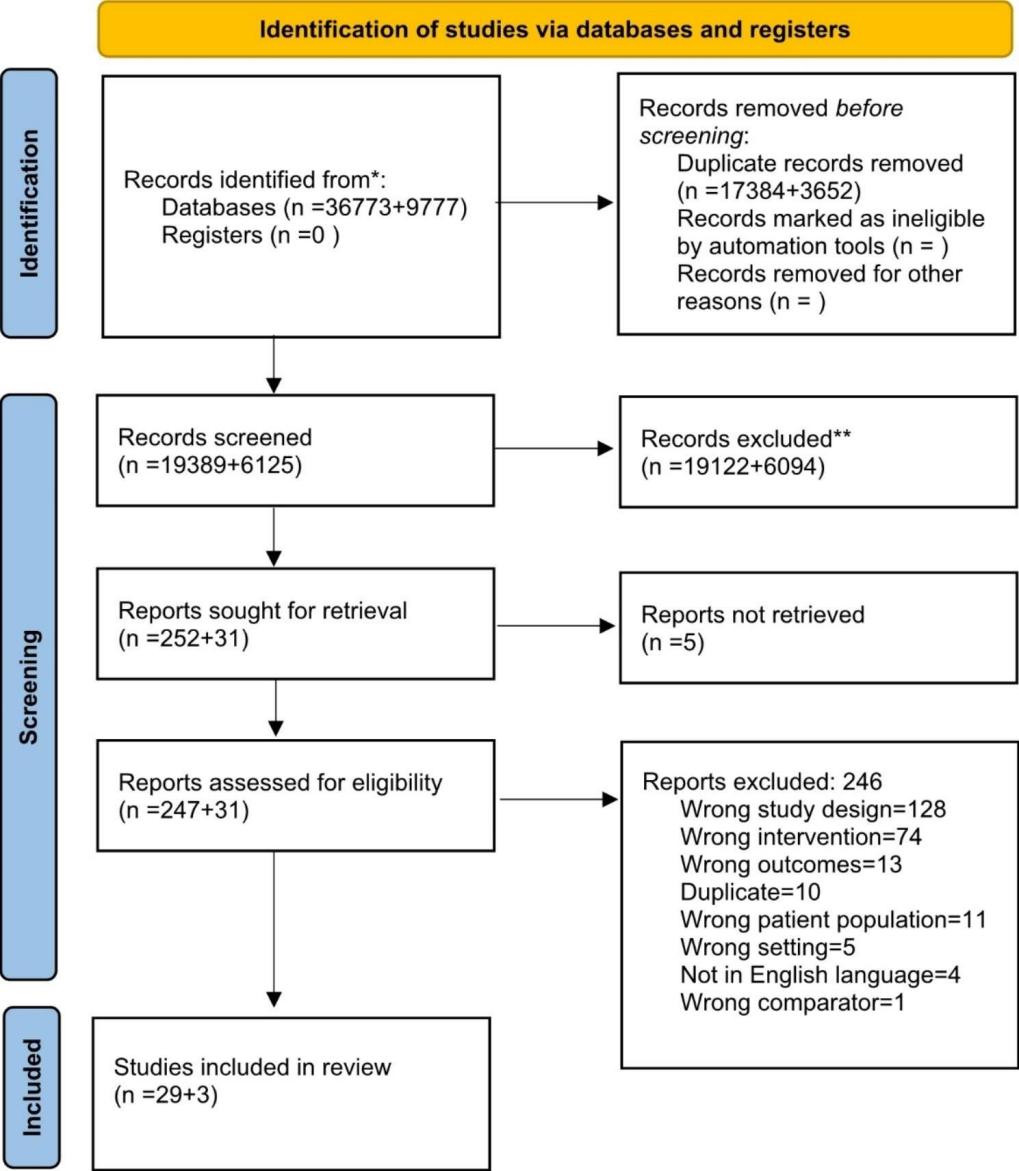
Prisma Flow diagram *Consider, if feasible to do so, reporting the number of records identified from each database or register searched (rather than the total number across all databases/registers) **If automation tools were used, indicate how many records were excluded by a human and how many were excluded by automation tools *From*: Page MJ, McKenzie JE, Bossuyt PM, Boutron I, Hoffmann TC, Mulrow CD, et al. The PRISMA 2020 statement: an updated guideline for reporting systematic reviews. BMJ 2021;372:n71. doi: 10.1136/bmj.n71 more information, visit: http://www.prisma-statement.org/

After the removal of duplicates, the initial search generated 25,514 citations. Titles and abstracts were screened, following which we undertook a full text screening of 278 manuscripts. Manuscripts that did not have a clear connection with the intervention and our pre-defined outcomes were excluded. 34 manuscripts were included in the final analysis (Fig. 1. Prisma flow diagram).

All but three manuscripts [18–20] described data from the United States of America. The three other jurisdictions were Hong Kong [19], Tunisia [20], and Turkey [18]. The characteristics of included manuscripts are presented in Tables 1, 2, 3, 4 and 5. The included studies contained information relevant for the outcomes delayed abortion [21–27], continuation of pregnancy [25, 26, 28–39], opportunity costs [19, 20, 22, 25–27, 40, 41], unlawful abortion [18, 19], anticipated family disharmony [27; 41–43], anticipated exposure to interpersonal violence or exploitation [22, 27, 41, 42, 44], anticipated reproductive coercion [41–43, 27], and system costs [45–50]. No evidence was identified linking the intervention to the outcomes self-managed abortion. All but one of the studies [18] considered the effects of TPAs that apply when minors seek abortion; that study considered ‘spousal consent’ requirements.

**Table 1:** Characteristics of included studies, Parental Involvement

Author/year	Country	Methods	Participants/data sources
Colman 2013	United States of America	Time series (n = not reported)	Multiple data sources; data on the rates of STIs, current parental involvement laws and socio-economic information.
Fuentes 2019	United States of America	Cross sectional (n = 889)	Data from the Abortion Patient Survey and the Abortion Provider Census, 2014.
Hasselbacher 2014	Illinois, United States of America	Qualitative, in-depth interviews (n = 30)	Minors seeking an abortion in a in a state that did not mandate parental involvement
Hung 2010	Hong Kong	Qualitative, in-depth interviews (n = 29)	Girls and women aged 13–24 with experience of abortion in their adolescent years.
Joyce, 2019	United States of America	Cohort - non-comparative (n = 43,594)	Multiple data sources; data on adolescent pregnancies from Center for Disease Control and Guttmacher Institute, and data on parental involvement laws
Kavanagh 2012	United States of America	Qualitative, in-depth interviews (n = 30)	Minors seeking an abortion at one of three clinics in a state that mandate parental involvement in abortion decisions
MacAfee, 2015	New England, United States of America	Cohort study - non-comparative (n = 373)	Data on all minors seeking abortions at Planned Parenthood clinics in three states, 2011–2012
Medoff 2010a	United States of America	Time series design (n = not reported)	Multiple data sources: Data on non-marital birthrates from Centers for Disease Control; economic data from the US Census of Population, 2003
Medoff 2010b	United States of America	Time series (n = not reported);	Multiple data sources: Data on abortion from the Guttmacher Institute; socio-economic data from State Reports of the U.S Census Bureau
Medoff 2010c	United States of America	Time series (n = not reported);	Multiple data sources; socio-economic data from the U.S Bureau of the Census and the Statistical Abstract of the U.S; abortion data from Guttmacher institute
Medoff 2010d	United States of America	Time series (n = not reported);	Multiple data sources; data on adolescent pregnancies from the Guttmacher Institute
Medoff 2012a	United States of America	Time series (n = not reported);	Multiple data sources: Data on abortion from the Guttmacher Institute; socio-economic data from State Reports of the U.S Census Bureau
Medoff 2012b	United States of America	Time series (n = not reported);	Multiple data sources: Socioeconomic data from US Census of Population Data; Association of Religion Data Archives, abortion data from Guttmacher Institute
Medoff 2014a	United States of America	Time series design (n = not reported)	Multiple data sources: abortion data from Centers for Disease Control and Guttmacher Institute; socio-economic data from Statistical Abstract of the Unites States.
Medoff 2014b	United States of America	Time series design (n = not reported)	Multiple data sources: data on pregnancy intentions from Centers for Disease Control; data on births from the US Vital Statistics Report.
Medoff 2014c	United States of America	Time series design (n = not reported)	Multiple data sources: data on unintended pregnancy from a previous publication; abortion data from the Guttmacher Institute
Medoff 2016	United States of America	Time series design (n = not reported)	Abortion data from Guttmacher Institute; data on unintended births from a previous publication
Myers 2017	United States of America	Cross sectional (n = 3142)	Multiple data sources; data on abortion access and rates, adolescent pregnancies, demographic and economic information, and state level policies
Ralph 2018	Illinois, United States of America	Cohort study - comparative (n = 1577)	Data on women aged 17–20 obtaining an abortion at one clinic, before and after implementation of a parental notification law
Ramesh 2016	Illinois, United States of America	Cohort study - comparative (n = 5505)	Minors obtaining a first trimester abortion at one health care facility, before and after the implementation of a parental notification law
Sen 2012	United States of America	Cross sectional (n = 5100)	Multiple data sources; Data on homicide deaths < 5 from Center for Disease Control and Prevention and National Center for Health Statistics, Multiple Cause of Deaths,1983 to 2002
Tosh 2015	United States of America	Cohort study - comparative (n = not reported)	Multiple data sources; State level data on adolescent birth rates and reproductive health laws from Guttmacher Institute
Wallace 2017	United States of America	Cross sectional (n = 3,948,761 all births during 2012 in US)	Multiple data sources; data from the National Center for Health Statistics on live births focusing on preterm births and low birth weight.

**Table 2:** Characteristics of Included Studies: Judicial Bypass

Author/year	Country	Methods	Participants/data sources
Coleman-Minahan 2019	Texas, United States of America	Qualitative, individual in-depth interviews (n = 20)	Adolescents aged 16–19 with experience of trying to obtain a judicial bypass
Coleman-Minahan 2020	Texas,United States of America	Qualitative individual in-depth interviews (n = 20)	Young women aged 16–19 who sought to obtain a judicial bypass before and after Texas restructured the judicial bypass process in 2016.
Friedman 2015	Ohio, United States of America	Cohort study – non-comparative (n = 55)	Data on pregnant minors presenting for a court ordered evaluation for judicial bypass of parental consent for abortion, over a three-year period
Kavanagh 2012	United States of America	Qualitative, individual in-depth interviews (n = 30)	Minors seeking an abortion at three healthcare facilities in a state that mandates parental consent notification
Maffi & Affes 2019	Tunis, Tunisia	Qualitative ethnographic mixed methods study (n = not reported)	Participant observations from one government hospital and three sexual and reproductive health clinics; review of medical files; one personal abortion provider experience; interviews with women and health care providers
Ralph 2021	Illinois, United States of America	Retrospective cohort study (n = 128)	Data from phone or in-person consultations between Illinois Judicial Bypass Coordination Project (JBCP) attorneys and minors who contacted JBCP between 2017 and 2019.

**Table 3:** Characteristics of Included Studies: Judicial bypass vs. parental consent

Author/year	Country	Methods	Participants/data sources
Altindag 2017	United States of America	Time series design (n = 2624)	Data on all abortions within one state between 2005 and 2014, of which 10% were obtained through judicial bypass
Janiak 2019	Massachusetts, United States of America	Cohort study (n = 2026)	Minors 17 and younger seeking abortion at three healthcare facilities between 2010 and 2016; 77% were obtained through parental consent and 23% through judicial bypass
Joyce 2010	Arkansas, United States of America	Time series design (n = 7463)	Data on abortions among minors aged 15–17, performed between 2001 and 2007

**Table 4:** Characteristics of Included Studies: Parental consent v parental notification

Author/year	Country	Methods	Participants/data sources
Chevrette 2015	United States of America	Time series design (n = 434,503)	Multiple data sources: Data on adolescents aged 15–19, giving birth in 2008 from Center for Disease Control and Prevention, abortion data from Guttmacher Institute
Joyce 2010	Arkansas, United States of America	Time series design (n = 7463)	Data on abortions among minors aged 15–17, performed between 2001 and 2007

**Table 5:** Characteristics of Included Studies: Spousal Consent

Author/year	Country	Methods	Participants/data sources
MacFarlane 2016	Turkey	Qualitative – in-depth and semi-structured interviews (n = 25)	Interviews with key informants (physicians, pharmacists, one lawyer, n = 14) and women with experience of abortion (n = 11)

23 manuscripts considered what they termed ‘parental involvement’ [45, 40, 43, 19, 29, 41, 25, 30–32, 47, 46, 33, 34, 48, 35–37, 26, 38, 49, 39, 50]. These manuscripts did not specify the precise form of parental involvement required by law in the study setting but did make clear that some kind of parental involvement was mandated by law. In other words, these studies related to situations where the pregnant person’s parent or guardian had an involvement in the abortion decision because the law required that, rather than being involved because the pregnant young person chose to involve them. The summary findings from these studies are outlined in Table 6 and the findings by study are outlined in Supplementary Table 1.

**Table 6:** Impacts of parental involvement requirements for abortion seekers

Outcome	Overall conclusion of evidence	Applicable Human Rights Standards	Conclusion evidence + Human Rights
Delayed abortion	Evidence from two studies relating to parental notification is unclear. Variation in findings may be due to the study setting or inadequate sample size. However, minors who must travel outside their community to obtain abortion care experience significant delays in receiving care.	TPAs engage states’ obligations to respect, protect and fulfil the rights to life and health (by taking steps to reduce maternal mortality and morbidity), and to equality and non-discrimination (because of disproportionate impact on vulnerable groups).	Delay is associated with increased maternal mortality and morbidity. As any delays associated with parental involvement laws have disproportionate impact on specific populations, these laws are associated with reduced enjoyment of the right to health, the right to life, and the right to equality and non-discrimination.
Continuation of pregnancy	14 studies across five sub-outcomes suggest that parental involvement laws for minors decrease abortion access and contribute to continuation of pregnancy. The relationship between parental involvement laws and unintended pregnancy and birth rates suggests that overall, parental involvement laws increase adolescent birth rates but do not reduce unintended pregnancy or births. When parental consent is associated with increased birth rates, there is a disproportionate impact on adolescents within specific populations (Black teens) and those engaged in cross-border travel.	TPAs engage states’ obligations to protect, respect and fulfil the right to health, the right to decide on the number and spacing of children, the right to privacy, and the right to equality and non-discrimination.	As parental involvement laws may be associated with a reduction in overall abortion rates and may decrease the number of abortions for minors, and as those impacts can disproportionately affect certain populations, these laws can impact negatively on the right to equality and non-discrimination, and may undermine the rights to health and to security of person.
Opportunity costs	Evidence from four studies suggests that parental involvement laws are associated with increased opportunity costs for minors including opportunity costs due to travel for abortion to states where parental consent or notification is not required. Variation in findings may be due to specific differences in study settings.	TPAs engage states’ obligations to protect, respect and fulfil the right to health by ensuring abortion regulation is evidence-based and proportionate, that where it is lawful abortion is safe and accessible.	As parental consent laws may lead to opportunity costs with implications for the enjoyment of human rights, these laws have negative impacts on the rights to health and to security of person.
Unlawful abortion	Evidence from one study suggests that parental consent laws may lead to unlawful abortion among minors.	TPAs engage states’ obligations to respect, protect and fulfil the rights to life and health (by taking steps to reduce maternal mortality and morbidity, and by protecting people from the physical and mental health risks associated with unsafe abortions).	As parental involvement laws may increase unlawful abortion, and where unsafe may be associated with maternal mortality and morbidity and with physical and mental health risks, parental involvement laws impact negatively on the right to life and the right to health.
Self-managed abortion (SMA)	No studies identified.	TPAs engage states’ obligations to respect, protect and fulfil the rights to life and health (by taking steps to reduce maternal mortality and morbidity, and by protecting people from the physical and mental health risks associated with unsafe abortions).	If TPAs result in recourse to SMA and if such SMA is unsafe, TPAs impact negatively on abortion seekers’ rights.
Anticipated family disharmony	Evidence from two studies indicate minors anticipate that involuntary disclosure of a pregnancy due to requirements for parental notification or consent may increase risk of family disharmony.	TPAs engage states’ obligations to protect abortion seekers, the right to privacy, and the right to health.	As compelled disclosure of abortion seeking may expose minors to family disharmony, this may have negative impacts on the right to health and the right to privacy.
Anticipated exposure to violence or exploitation	Evidence from one study demonstrates that minors anticipate that involuntary disclosure of a pregnancy due to a requirement for parental notification may increase the risk for physical and psychological violence directed at them or their future children.	TPAs engage states’ obligations to protect abortion seekers, the right to privacy, and the right to health.	As compelled disclosure of abortion seeking may expose minors to violence, parental involvement laws impact negatively on rights to health and privacy, and engage states’ positive obligations to protect abortion seekers from harm.
Anticipated reproductive coercion	Evidence from two studies indicate that minors anticipate that involuntary disclosure of a pregnancy due to a requirement of parental notification or consent, may increase the risk for reproductive coercion.	TPAs engage states’ obligations to protect the right to health, right to security of person, right of persons with disabilities to retain fertility on an equal basis with others, right to be free from torture, and cruel, inhuman or degrading treatment, right to exercise legal capacity, right to decide on the number and spacing of children, right to equality and non-discrimination, right to privacy, and women’s right to legal capacity on an equal basis with men.	As compelled disclosure of abortion seeking can expose minors to reproductive coercion, these laws may impact negatively on the right to health, right to security of person, right of persons with disabilities to retain fertility on an equal basis with others, right to be free from torture, and cruel, inhuman or degrading treatment, right to exercise legal capacity, the right to decide on the number and spacing of children, right to equality and non-discrimination right to privacy, and women’s right to legal capacity on an equal basis with men; engaging states positive obligation to protect abortion seekers from harm and from forced or coerced abortion.
System costs	Overall evidence from six studies across six sub-outcomes suggest that parental involvement laws increase system costs. Parental involvement laws have no impact on STI cases or pregnancy rates. Parental involvement laws may increase system costs related to pre-term birth and low birth weight, unwanted pregnancy rates and child homicide deaths.	TPAs engage states’ obligations to protect, respect and fulfil the right to health (by ensuring that where it is lawful abortion is safe and accessible, and that regulation of abortion is evidence-based and proportionate).	As parental involvement laws may increase and not reduce system costs, these laws have negative impacts on the rights to health and to security of person.

Six manuscripts considered ‘judicial bypass’ [20, 22, 27, 41, 42, 44], while a further three studies considered comparatively the impacts of judicial bypass and ‘parental consent’ [21, 23, 24]. The summary findings from these studies are outlined in Tables 7 and 8 respectively, with the findings by study being contained in Supplementary Tables 2 and 3 respectively.

**Table 7:** Impact of judicial bypass requirements on abortion seekers

Outcome	Overall conclusion of evidence	Applicable Human Rights Standards	Conclusion evidence + Human Rights
Delayed abortion	Evidence from two studies suggests that judicial bypass may be associated with delayed abortion.	TPAs engage states’ obligations to respect, protect and fulfil the rights to life and health (by taking steps to reduce maternal mortality and morbidity), and to equality and non-discrimination (because of disproportionate impact on vulnerable groups).	Delay is associated with increased maternal mortality and morbidity. As these delays apply only to minors without regard to their individual capacity to consent to medical treatment, judicial bypass is associated with reduced enjoyment of the right to health, the right to life, and the right to equality and non-discrimination.
Continuation of pregnancy	No studies identified.	TPAs engage states’ obligations to protect, respect and fulfil the right to health, the right to decide on the number and spacing of children, the right to privacy, and the right to equality and non-discrimination.	If judicial bypass is associated with unwanted continuation of pregnancy, this would impact negatively on the right to equality and non-discrimination, the right to health, and the right to security of person.
Opportunity costs	Evidence from four studies supports that judicial bypass may be associated with opportunity costs Some minors need a confidential pathway to obtain abortion care. These minors report meaningful logistical burdens and opportunity costs in obtaining an abortion by judicial bypass.	TPAs engage states’ obligations to protect, respect and fulfil the right to health by ensuring abortion regulation is evidence-based and proportionate, and that where it is lawful abortion is safe and accessible.	As judicial bypass is associated with increased opportunity costs compared to a minor’s own ability to consent, these mechanisms may be associated with reduced enjoyment of the right to health, the right to security of person, and the right to equality and non-discrimination.
Unlawful abortion	No studies identified.	TPAs engage states’ obligations to respect, protect and fulfil the rights to life and health (by taking steps to reduce maternal mortality and morbidity, and by protecting people from the physical and mental health risks associated with unsafe abortions).	If judicial bypass results in recourse to unlawful abortion it impacts negatively on abortion seekers’ rights to life, health, and equality and non-discrimination.
Self-managed abortion (SMA)	No studies identified.	TPAs engage states’ obligations to respect, protect and fulfil the rights to life and health (by taking steps to reduce maternal mortality and morbidity, and by protecting people from the physical and mental health risks associated with unsafe abortions).	If judicial bypass results in recourse to SMA and if such SMA is unsafe, judicial bypass impacts negatively on abortion seekers’ rights
Anticipated exposure to interpersonal violence or exploitation	Evidence from four studies indicates that minors value and need a pathway to obtain confidential abortions. Minors request judicial bypass when they anticipate violence if a pregnancy is disclosed. Judicial bypass may decrease anticipated violence by creating a pathway where minors can obtain confidential abortions.	TPAs engage states’ obligations to protect abortion seekers, the right to privacy, and the right to health.	Where parental authorization/notification/involvement laws exist, judicial bypass can provide an alternative route to accessing abortion that reduces anticipated exposure to interpersonal violence or exploitation. Thus, judicial bypass may enhance enjoyment of the right to health and the right to privacy *relative to* parental consent requirements. However, the requirement for judicial authorization impacts negatively on the right to privacy when compared to the ability to consent to medical treatment according to capacity.
Anticipated reproductive coercion	Evidence from three studies indicates that minors value and need a pathway to obtain confidential abortions. Minors request judicial bypass when they anticipate reproductive coercion if a pregnancy is disclosed. Judicial bypass may decrease the risk of reproductive coercion by creating a path where minors can have confidential abortions.	TPAs engage states’ obligations to protect abortion seekers, the right to privacy, and the right to health.	Where parental authorization/notification/involvement laws exist, judicial bypass can provide an alternative route to accessing abortion that reduces anticipated reproductive coercion, Thus, judicial bypass may enhance enjoyment of the right to health, right to security of person, right of persons with disabilities to retain fertility on an basis with others, right to be free from torture, and cruel, inhuman or degrading treatment, right to exercise legal capacity, women’s right to legal capacity on an equal basis with men *relative to* parental consent requirements. However, the requirement for judicial authorization impacts negatively on the right to decide on the number and spacing of children, right to equality and non-discrimination, and right to privacy.
Anticipated family disharmony	Evidence from three studies indicates that minors value and need a pathway to obtain confidential abortions. Minors request judicial bypass when they anticipate family disharmony if a pregnancy is disclosed. Judicial bypass may decrease family disharmony by creating a path where minors can have confidential abortions.	TPAs engage states’ obligations to protect the right to health, right to security of person, right of persons with disabilities to retain fertility on an equal basis with others, right to be free from torture, and cruel, inhuman or degrading treatment, right to exercise legal capacity, right to decide on the number and spacing of children, right to equality and non-discrimination, right to privacy, and women’s right to legal capacity on an equal basis with men.	Where parental authorization/notification/involvement laws exist, judicial bypass can provide an alternative route to accessing abortion that reduces anticipated family disharmony. Thus, judicial bypass may enhance enjoyment of the right to health *relative to* parental consent. However, the requirement for judicial authorization impacts negatively on the right to privacy.
System costs	No studies identified.	TPAs engage states’ obligations to protect, respect and fulfil the right to health (by ensuring that where it is lawful abortion is safe and accessible, and that regulation of abortion is evidence-based and proportionate).	If judicial bypass increases system costs, it may have negative impacts on the right to health.

**Table 8:** Judicial bypass vs. parental consent: Impact on abortion seekers

Outcome	Overall conclusion of evidence	Applicable Human Rights Standards	Conclusion evidence + Human Rights
Delayed abortion	Evidence from three studies examining the difference between judicial bypass and parental consent on delayed abortion is unclear. Differences in estimates may be due to significant variation in the bypass process across settings. When judicial bypass is associated with greater delays compared with parental consent, minors using judicial bypass are more likely to pass gestational thresholds for medical abortion per local guidance. Evidence from two studies suggest that specific populations of minors are more likely to use judicial bypass than parental consent to obtain an abortion, and thus may be disproportionately impacted by the effects of judicial bypass.	TPAs engage states’ obligations to respect, protect and fulfil the rights to life and health and to equality and non-discrimination (because of disproportionate impact on vulnerable groups).	Delays are associated with increased maternal mortality and morbidity. As judicial bypass may be used more by specific populations and may be associated with increased delay, judicial bypass is associated with reduced enjoyment of the right to health, the right to life, and the right to equality and non-discrimination.
Continuation of pregnancy	No studies identified.	--	
Opportunity costs	No studies identified.	--	
Unlawful abortion	No studies identified.	--	
Self-managed abortion	No studies identified.	--	
Anticipated exposure to violence or exploitation	No studies identified.	--	
Anticipated reproductive coercion	No studies identified.	--	
Anticipated family disharmony	No studies identified.	--	
System costs	No studies identified.	--	

Two included studies [28, 24] compared the impacts of parental consent and parental authorization and notification requirements (Supplementary Tables 4 and 5). Finally, one of the included studies considered the impacts of ‘spousal consent’ requirements [18] (Supplementary Tables 6 and 7).

### Delayed abortion

Two studies [25, 26] found that parental notification laws per se are not associated with increased gestational age among minors seeking abortion, however one of these studies showed that minors who must travel outside of their community to access abortion care without a TPA experience a higher proportion of second trimester abortions compared to that in abortion seekers aged 18–21 [26]. Two studies showed that minors who use judicial bypass (i.e., who seek to circumvent parental authorization requirements) do experience delayed abortion [22, 27], but the three studies that compared judicial bypass to parental consent requirements presented a mixed picture about the comparative delays between the two systems [21, 23, 24]. One study suggested that using judicial bypass resulted in greater delays than the parental consent requirement and that minors using judicial bypass are more likely to pass gestational limits that render them ineligible for medical abortion [23], while two suggested that judicial bypass resulted in shorter delays than where the parental consent requirement is fulfilled [21, 24]. Two studies suggested that judicial bypass is especially important for subpopulations of minors, namely those coming from a minority racial [23, 24], or lower socioeconomic background [23], those under 15 [24], and those who are resident out of state [24]. The one study that compared parental consent to parental notification found that there was no difference in rates of second semester abortion between them [24]. While this presents a somewhat mixed picture, it does suggest that in at least some cases TPAs are associated with delays to abortion and that such delays may even be such as to result in an abortion seeker exceeding a gestational limit and thus becoming ineligible for lawful abortion. Delayed abortion may in some cases be more complex or intrusive than early abortion raising the possibility of potential increased maternal mortality or morbidity. This is notwithstanding the fact that states are strictly required to take steps to reduce and prevent maternal mortality or morbidity as a matter of international human rights law [2, 8], particularly for adolescent girls [7].

### Continuation of pregnancy

The studies considered in this review suggested that TPA requirements are associated with decreased access to abortion and contribute to the continuation of pregnancy. When considered by reference to abortion rates, three studies found that parental involvement requirements were associated with reduced overall abortion rates for minors and adults [31–33], while two found that parental notification requirements are associated with a decrease in the number of abortions among minors [25, 26]. Three further studies suggested either that parental involvement laws had no impact on the number of abortions among minors [29, 39] or that any such impact was unclear [38]. One study showed that parental involvement laws are not associated with reductions in overall unintended pregnancy rates among minors and adults [35], while another showed that they are not associated with increasing unintended birth rates [36]. However, three studies [30, 37, 39] showed that parental involvement laws are associated with an increase in birth rates among adolescents, so that reduced abortion rates can be interpreted as suggesting an increase in continuation of pregnancy. One study suggests that states with parental consent laws have lower abortion rates but no difference in adolescent birth rates to those where parental notification or no TPA are mandated [28]. Although one study suggested that parental consent laws do not have a different effect on rates of abortion between white, Black or Hispanic women [34], another showed that increased birth rates associated with parental consent laws are disproportionately experienced by Black minors and those who must travel over 100 miles to reach another US state where no parental TPA applies [37]. The evidence from these studies indicates that TPAs engage states’ obligations to protect the right to health (which requires that sexual and reproductive healthcare be available, accessible, acceptable and of quality [1], including legal and safe abortion care [6]), the right to privacy, and the right to decide the number and spacing of children. As abortion restrictions only apply to women, and as TPAs can be applicable only to sub-categories of abortion seekers, the right to equality and non-discrimination is also implicated [51] including where, as suggested by the evidence from included studies, the TPA requirements impact disproportionately on sub-populations of pregnant people.

### Opportunity costs

Evidence from four studies suggests that TPAs are associated with opportunity costs for those who seek abortion [19, 25, 26, 40]. While two studies found that there was no increase in inter-state travel to obtain abortion care where parental notification requirements are implemented [25, 26], two studies suggested that travel-related opportunity costs are associated with parental TPAs. Evidence from one study showed that, where parental notification laws apply, minors are more likely to travel long distances to access abortion [40], and another (based in Hong Kong) showed that some minors will travel to obtain unlawful abortion to avoid parental consent laws [19]. Judicial bypass procedures are also associated with opportunity costs for minors, including logistical burdens (like time missed from school, work, and home), uncertainty and delays while decisions about the application are being made [20], and travel to and from court [22, 27]. In one study, minors reported that the need for judicial bypass would complicate access to abortion significantly because of logistical burdens and difficulties in finding free or affordable legal services [41]. As already mentioned, satisfaction of the right to the highest attainable standard of physical and mental health requires states to ensure that healthcare—including sexual and reproductive healthcare—is available, accessible, acceptable and of quality [1]. Opportunity costs of the kind demonstrated in the included studies reduce the accessibility of abortion care, thus undermining the right to health.

### Unlawful abortion

Evidence from one study suggested that minors resort to unlawful abortion in order to avoid parental consent laws [19]. A further study suggested that where spousal TPA requirements are in place, some women will resort to unlawful abortion to avoid them [18]. While unlawful abortion is not always unsafe, it is generally *less* safe [52] (i.e., it meets only one of the two conditions for safe abortion: provided both by an appropriately trained provider and using a recommended method). As a matter of international human rights law, states are obliged to protect abortion seekers from, and to take steps to reduce, unsafe abortion [3]. The evidence from these included studies suggests that TPAs operate contrary to these obligations.

### Anticipated exposure to interpersonal violence or exploitation

Several studies included in this review suggest an association between TPA requirements and abortion seekers’ anticipated exposure to interpersonal violence or exploitation. One study showed that minors are concerned that parental notification laws will expose them (and in some cases their future children) to physical or psychological violence during or after pregnancy [41]. As states are required to protect abortion seekers, including ensuring that lawful abortion is effectively available without adverse consequences for those seeking it [53], associations between TPAs and exposure to violence or exploitation raise significant questions of human rights compliance. Four studies showed that, where parental TPAs are required, minors use judicial bypass to avoid anticipated violence [22, 27, 42, 44], suggesting that judicial bypass may be a rights-enhancing measure in the context of TPA requirements. However, the existence of judicial bypass is itself a product of the TPA requirement so that even if it mitigates, it likely cannot alleviate the human rights implications arising from the TPA.

### Anticipated reproductive coercion

Evidence from two studies showed that minors are concerned that parental TPA requirements will diminish their reproductive autonomy and put them at risk of forced abortion or forced continuation of pregnancy [41, 43]. Either outcome violates the pregnant person’s rights. Non-consensual abortion is a serious human rights violation that may constitute cruel, inhuman, or degrading treatment or punishment [54, 55] and violates the right to health [56]. Accordingly, states are required to take steps to prevent it [6, 57]. Forced or coerced continuation of pregnancy also results in human rights violations, which may include a violation of the right to decide on the number and spacing of children in Article 16(1) of the Convention on the Elimination of all forms of Discrimination Against Women (CEDAW). The CEDAW Committee has made it clear that decisions whether to have children or not, while preferably made in consultation with a spouse or partner, must not be limited by spouse, parent, partner, or Government; they are for the pregnant person herself to make [11]. In respect particularly of adolescents, multiple human rights actors have made clear states’ obligations to ensure health systems and services can meet the specific sexual and reproductive health needs of adolescents [7], including having access to non-discriminatory, confidential, and responsive sexual and reproductive healthcare including safe abortion services [69]. According to three included studies, avoiding reproductive coercion is one reason minors report for not wanting to disclose their pregnancy to a parent [43] and for using judicial bypass [27, 42]. Thus, judicial bypass may once more mitigate the human rights harms of other TPA requirements, but it too is a TPA and thus constitutes a non-rights-based barrier to access to quality abortion.

### Anticipated family disharmony

Evidence from two studies shows that minors anticipate that the involuntary disclosure of their pregnancy due to TPA requirements would lead to profound change in their relationship with their parent [41, 43]. Minors reported that seeking to avoid such disharmony within their family was a reason for not disclosing a pregnancy to a parent [43] and for seeking to use judicial bypass instead [27, 42].

### System costs

Five of the studies included in this review suggest that TPAs are associated with either increasing or no additional system costs. Evidence from one study shows that parental involvement laws are not associated with increased rates of sexually transmitted infections [45] another that they are not associated with increased rates of adolescent pregnancies [47], and another that they are not associated with increased occurrence of postpartum depression [48]. However, one study showed that parental involvement laws are associated with increased odds of preterm birth and low birth weight infants [50], and another with increased numbers of homicide-related deaths in children under five [49]. Only one study suggested an association with decreased system costs, in this case showing an association between parental involvement laws and an overall reduction in unintended pregnancy rates among both minors and adults [46].

## Discussion

TPA requirements are aberrations from the usual principles of consent to health care interventions outlined in the introductory section of this review. Although predominantly focused on one class of persons (minors) and in one setting (the United States of America), the studies included in this review underline what human rights bodies have long made clear about TPAs: that they infringe on privacy and confidentiality and on the right to the highest attainable standards of physical and mental health, and that they operate as barriers to access to healthcare rather than as modes of supporting a pregnant person’s decision-making [2, 3, 51, 58]. This is similarly true of measures introduced purportedly as mitigations for other TPA requirements (such as judicial bypass as an alternative to parental consent requirements), which themselves operate as modes of TPA. In this respect, it is worth recalling the basis for the general principle- that it is a healthcare seeker alone whose consent is required for a health intervention.

Adolescents are frequently the objects of TPA requirements [10]. Such TPAs are rooted in a set of stereotypical and often patriarchal assumptions about young people that fail to reflect the well-established requirements of international human rights law, but which are widely reflected in health laws on adolescent consent and privacy [59]. Children have a right to freely express their views in all matters affecting them, including their reproductive lives and health care, under Article 12, UN Convention on the Rights of the Child. Consistent with the principle of recognizing young people’s evolving capacity, Article 12 makes clear that young people’s views must always be given due weight according to their age and maturity. Importantly, maturity cannot be determined based on chronological age alone. Rather, the UN Committee on the Rights of the Child has made clear that maturity should be understood as the “capacity of a child to express her or his views on issues in a reasonable and independent manner”. As a general principle, “the greater the impact of the outcome on the life of the child, the more relevant the appropriate assessment of the maturity of that child” [60, para 30]. Decisions about the continuation of pregnancy clearly have a profound impact on the young person’s life, not only because of the physical and emotional effects of pregnancy per se but also because unintended pregnancy and childbearing can impact significantly on educational attainment, economic opportunities, and ability to participate fully in public life [61, 62].

Accordingly, the UN Committee on the Rights of the Child has consistently emphasized that adolescents’ preferences in the context of abortion must be respected [63, para 66(b); 64, para 46]. Adolescents have a right to access “confidential adolescent-responsive and non-discriminatory reproductive and sexual health information and services, available both on and off-line” [65, para 59]. The UN Committee on the Rights of the Child has specifically called for adolescents to have access to confidential abortion services [66, 67]. Healthcare providers have an obligation to maintain young people’s confidentiality; they may reveal confidential medical information only with the adolescent’s consent.

For some this raises a dilemma. How can law and policy adequately support young people in forming decisions about their lives, including with the support of their parents and loved ones, while also respecting their human rights? International human rights bodies and experts have offered considerable guidance in this respect. As confirmed by the UN Committee on the Rights of the Child, states must review their legislation to guarantee the best interests of adolescents and ensure their views are always heard and respected in abortion-related decisions [65, para 60], and take steps to ensure that girls can make autonomous and informed decisions on their reproductive health [68, para 56]. The Committee has called on states to consider introducing a legal presumption that adolescents are competent to seek and have access to preventive or time-sensitive sexual and reproductive health commodities and services, [65, para 39], a suggestion that has been endorsed by the Special Rapporteur [69]. In practice this would mean that health workers are under no obligation to notify parents or otherwise seek to involve anyone other than the young person, although if the young person voluntarily seeks support from a third party—including a parent—in her decision-making about whether to continue with her pregnancy that can be facilitated. Any obligation to involve a third party would arise only if the health worker were to conclude, based on an individual assessment of the abortion seeker’s maturity and understanding, that she does not have capacity to reach this decision without such support. Even in such cases, a rights-based approach would comprise of seeking to support the young person in making a decision and *not* in substituting the decision of a parent, guardian, court or other authority for hers.

Although the studies considered in this review focused primarily on the application of TPA requirements relating to adolescents, there is a well-developed body of international human rights law that makes clear that TPA requirements are similarly rights-infringing in other contexts. The UN Human Rights Committee has said that requiring judicial authorization violates the right to privacy because it seeks to resolve through judicial proceeding what should be resolved between a health provider and the person who seeks abortion [70]. Furthermore, people have a right to decide for themselves on the number and spacing of children [71, Article 16(1)]. While human rights bodies have recognized that it may in principle be desirable for such a decision to be made in consultation with a spouse or partner, that decision must not be *limited by* spouses, parents, partners, governments [62] or health authorities [11]. Any barrier to accessing lawful abortion that is not based on medical need has been deemed discriminatory by the UN Working Group on the issue of Discrimination against Women in Law and in Practice [51]. As TPA requirements apply to categories of women (i.e., those who are pregnant) seeking a particular type of health care (i.e., abortion) without regard to their mental capacity to consent to health interventions, they are properly understood as discriminatory barriers to accessing sexual and reproductive health care.

## Limitations

This review has its limitations. As already mentioned, the included studies are disproportionately from the United States of America and limited to manuscripts published in English. TPAs are by no means particular to the United States of America and are widely found in national and sub-national abortion laws, which are largely not represented in the review [10]. Similarly, the included studies are almost all concerned with the impact of TPAs on minors, with limited data on the impact of other forms of TPA. This reflects a need for further research into the impacts of other forms of TPAs in abortion law and policy. As regards TPAs applying to minors, the included studies did not explain fully how TPAs interact in practice with general principles and practices relating to consent to healthcare interventions for young people and their developing capacity. Accordingly, further research on how TPA requirements interact with general laws or policies relating to consent to healthcare interventions would be welcome. Randomized controlled trials or comparative observational studies are not appropriate to studies that seek to understand the human rights implications of abortion-related interventions. In this field, studies are often conducted without comparisons. While this may be considered a limitation from a standard methodological perspective for systematic reviews, it does not limit the ability to identify human rights law implications of TPAs or similar interventions in law and policy and thus does not operate as a limitation within the context of the integrated methodology used here [13]. Standard tools for assessing risk of bias or quality, including GRADE [72] or the use of plausibility as an inclusion criterion, are not suited to a review that aims fully to integrate human rights implications into our understanding of the effects of TPAs as a regulatory intervention and thus engages with a wide variety of sources. Finally, in line with the methodology approach applied[13], this review applies international human rights law, rather than regional or domestic human rights law. While this enabled us to develop a general understanding of the rights-related implications of TPAs, multiple factors (including a state’s ratification of human rights instruments and their status in domestic law) will determine the applicability of any individual human rights standard in a specific setting [16, p. 7].

## Conclusion

This review synthesizes legal and health evidence to demonstrate the negative health and human rights impacts of TPA requirements on abortion seekers. It provides further substantiation of the pre-existing position in international human rights law that any requirements that abortion be authorized by third parties like parents, spouses, committees, and courts create barriers to abortion should not be introduced at all, or should be repealed where they exist. The review thus establishes that rights-based regulation of abortion should not impose TPAs in any circumstances. Instead, the provision and management of abortion should be treated in a manner cognizant with the general principles of informed consent in international human rights law, presuming capacity in all adults regardless of marital status and treatment sought, and recognizing the evolving capacity of young people in line with their internationally-protected rights.

### Electronic supplementary material

Below is the link to the electronic supplementary material.

Supplementary Material 1

## Data Availability

All data relevant to the study are included in the article or uploaded as online supplemental information.

## References

[1] General Comment CESCR. No. 14 (2000) The Right to the Highest Attainable Standard of Health (Art. 12 of the International Covenant on Economic, Social and Cultural Rights). UN Doc. E/C.12/2000/4, (11 August 2000).

[2] CESCR, General Comment No. 22 (2016) on the right to sexual and reproductive health (article 12 of the International Covenant on Economic, Social and Cultural Rights). UN Doc. E/C.12/GC/22, (2 May 2016).

[3] General comment HRC No. 36 (, Doc UN. CCPR/C/GC/36, (30 October 2018), Advance unedited version.

[4] UNESCO., 2005, Universal Declaration on Bioethics and Human Rights, 19 October 2005. 17139812

[5] WHO., 1994, A Declaration on the Promotion of Patients’ Rights in Europe, European Consultation on the Rights of Patients: Amsterdam 28–30 March 1994. ICP/HLE 121, 28 June 1994.

[6] Special Rapporteur on the right. Of everyone to the enjoyment of the highest attainable standard of physical and mental health, “Right of everyone to the enjoyment of the highest attainable standard of physical and mental health: report of the Special Rapporteur on the right of everyone to the enjoyment of the highest attainable standard of physical and mental health”, UN Doc. A/64/272, (10 August 2009).

[7] CRC, General comment No. 4. (2003): Adolescent Health and Development in the Context of the Convention on the Rights of the Child, UN Doc. CRC/GC/2003/4, (1 July 2003).

[8] (2014): Article 12: Equal recognition before the law, CRPD, General comment No. 1, Doc UN. CRPD/C/GC/1, (19 May 2014).

[9] UN., Convention on the Rights of Persons with Disabilities, UN Doc. A/RES/61/106, (19 December 2006).

[10] Lavelanet A, Johnson BR, Ganatra B (2020). Global abortion policies database: a descriptive analysis of the regulatory and policy environment related to abortion. Best Pract Res Clin Obstet Gynaecol.

[11] CEDAW, General Recommendation No. 24: Article 12 of the Convention (Women and Health). UN Doc. A/54/38/Rev.1, chap. I., (1999).

[12] UN, Convention on the Rights of the Child., UN doc. E/CN.4/RES/1990/74., (1990).

[13] de Londras F, Cleeve A, Rodriguez MI., Integrating rights and evidence: a technical advance in abortion guideline development, BMJ Glob Health, 2021;6; e004141. 10.1136/bmjgh-2020-004141 PMC7871678 33558339

[14] Petticrew M, Knai C, Thomas J. Implications of a complexity perspective for systematic reviews and guideline development in health decision making, BMJ Glob Health, 2019;4;e000899. 10.1136/bmjgh-2018-000899 PMC6350708 30775017

[15] Thomson HJ, Thomas S (2013). The effect direction plot: visual display of non-standardised effects across multiple outcome domains. Res Synth Methods.

[16] WHO, Guideline AC. World Health Organization: Geneva, (2022).

[17] Burris S, Ghorashi AR, Cloud LF et al. Identifying data for the empirical assessment of law (IDEAL): a realist approach to research gaps on the health effects of abortion law, BMJ Glob Health, 2021;6;e005120. 10.1136/bmjgh-2021-005120 PMC8202112 34117010

[18] MacFarlane KA, O’Neil ML, Tekdemir D (2016). Politics, policies, pronatalism, and practice: availability and accessibility of abortion and reproductive health services in Turkey. Reprod Health Matters.

[19] Hung SL (2010). Access to safe and legal abortion for teenage women from deprived backgrounds in Hong Kong. Reprod Health Matters.

[20] Maffi I, Affes M (2019). The right to abortion in Tunisia after the Revolution of 2011: legal, Medical, and social arrangements as seen through seven abortion stories. Health Hum Rights J.

[21] Altindag O, Joyce T (2017). Judicial bypass for Minors seeking abortions in Arkansas Versus Other States. Am J Public Health.

[22] Coleman-Minahan K, Stevenson AJ, Obront E (2019). Et. al., Young Women’s Experiences obtaining judicial bypass for abortion in Texas. J Adolesc Health.

[23] Janiak E, Fulcher IE, Cottrill AA (2019). Et. al., Massachusetts’ parental consent law and procedural timing among adolescents undergoing abortion. Obstet Gynecol.

[24] Joyce T (2010). Parental consent for abortion and the judicial bypass option in Arkansas: Effects and Correlates. Perspect Sex Reprod Health.

[25] MacAfee L, Castle J, Theile RN (2016). Association between the New Hampshire parental notification Law and Minors undergoing abortions in Northern New England. Obstet Gynecol.

[26] Ralph LH, King E, Belusa E. The impact of a parental notification requirement on Illinois Minors’ Access to and decision-making around abortion, J Adolesc Health, 2018;62l;281–287. 10.1016/j.jadohealth.2017.09.031 29248391

[27] Ralph LJ, Chaiten L, Werth E (2021). Reasons for and logistical Burdens of Judicial Bypass for abortion in Illinois. J Adolesc Health.

[28] Chevrette M, Abenhaim HA (2015). Do state-based policies have an impact on Teen Birth Rates and Teen Abortion Rates in the United States?. J Pediatr Adolesc Gynecol.

[29] Joyce T, Kaestner R, Ward J. The Impact of Parental Involvement Laws on Minor Abortion, 2019 *NBER Working Paper Series, Working Paper* 25758.

[30] Medoff MH (2010). Nonmarital births and state abortion policies. Soc Work Pub Health.

[31] Medoff MH (2010). State Abortion Policy and the long-term impact of parental involvement laws. Pol Pol.

[32] Medoff MH (2010). State abortion policies, targeted regulation of abortion provider laws, and abortion demand. Rev Pol Res.

[33] Medoff MH, Unintended Pregnancies RA, Laws, Demand A. Int Schol Res Net, 2012;Article ID 612081.

[34] Medoff MH (2014). Race, restrictive state abortion laws and abortion demand. Rev Black Polit Econ.

[35] Medoff MH, Unintended Pregnancy and State Abortion Policy (2014). Rational choice or Random Behavior. J Pol Prac.

[36] Medoff MH (2016). State Abortion Policy and Unintended Birth Rates in the United States. Soc Indic Res.

[37] Myers CK, Ladd D. Did Parental Involvement Laws Grow Teeth? The Effects of State Restrictions on Minors’ Access to Abortion, 2017 *IZA Discussion Papers* No. 10952. 10.1016/j.jhealeco.2020.102302 32135395

[38] Ramesh S, Zimmerman L, Patel A (2016). Impact of parental notification on Illinois Minors seeking abortion. J Adolesc Health.

[39] Tosh J. State Adolescent Reproductive Health Policies and their Impact on Teen Pregnancy Outcomes, PhD thesis: University of Florida (2015). Available at https://stars.library.ucf.edu/cgi/viewcontent.cgi?article=1058&context=etd

[40] Fuentes L, Jerman J (2019). Distance traveled to Obtain Clinical Abortion Care in the United States and reasons for Clinic Choice. J Women’s Health.

[41] Kavanagh EK, Hasselbacher LA, Betham B (2012). Abortion-seeking Minors’ views on the Illinois parental notifi cation law: a qualitative study. Perspect Sex Reprod Health.

[42] Coleman-Minahan K, Stevenson AJ, Obront E (2020). Et. al., adolescents obtaining abortion without parental consent: their reasons and experiences of Social Support. Perspect Sex Reprod Health.

[43] Hasselbacher LA, Dekleva A, Tristan S (2014). Et. al., factors influencing parental involvement among Minors seeking an abortion: a qualitative study. Am J Public Health.

[44] Friedman SH, Hendrix T, Haberman J (2015). Et. al, Judicial bypass of parental consent for abortion: characteristics of pregnant minor “Jane Doe’s. J Nerv Ment Dis.

[45] Colman S, Dee TS, Joyce TJ. Do parental involvement laws deter risky teen sex?, 2013 *NBER Working Paper Series*, Working Paper 18810. 10.1016/j.jhealeco.2013.06.003 23892483

[46] Medoff MH. Unintended pregnancy and abortion Access in the United State, Int J Pop Res. 2012;Article ID 254315.

[47] Medoff MH (2010). The impact of State Abortion policies on teen pregnancy rates. Soc Indic Res.

[48] Medoff MH (2014). The relationship between restrictive state abortion laws and Postpartum Depression. Soc Work Public Health.

[49] Sen B, Wingate MS, Kirby R (2012). The relationship between state abortion-restrictions and homicide deaths among children under 5 years of age: a longitudinal study. Soc Sci Med.

[50] Wallace ME, Evans MG, Theall K (2017). The status of women’s Reproductive Rights and adverse birth outcomes. Women’s Health Issues.

[51] HRC, Report of the Working Group on the issue of discrimination against women in law and in practice, UN Doc A/HRC/32/44, (8 April 2016).

[52] Ganatra B, Gerdts C, Rossier C (2017). Global, regional, and subregional classification of abortions by safety, 2010–14: estimates from a bayesian hierarchical model. Lancet.

[53] Report of the Special Rapporteur on torture and other cruel, inhuman or degrading treatment or punishment, Méndez JE. UN Doc. A/HRC/22/53, (1 February 2013).

[54] CEDAW, General recommendation No. 35 on gender-based violence against women, updating general recommendation No. 19 UN Doc. CEDAW/C/GC/35, (26 July 2017).

[55] CRPD, General comment No. 3. (2016) on women and girls with disabilities. UN Doc. CRPD/C/GC/3, (25 November 2016).

[56] Special Rapporteur. 2011, Right of everyone to the enjoyment of the highest attainable standard of physical and mental health: interim report of the Special Rapporteur on the right of everyone to the enjoyment of the highest attainable standard of physical and mental health, UN Doc. A/66/254, (3 August 2011).

[57] General HRC. comment no. 28 (68), Equality of rights between men and women (article 3): General comments adopted by the Human Rights Committee under article 40, paragraph 4, of the International Covenant on Civil and Political Rights: addendum, UN Doc. CCPR/C/21/Rev.1/Add.10, (29 March 2000).

[58] (2016) on the rights of rural women, CEDAW, General recommendation No. 34, Doc UN. CEDAW/C/GC/34, (7 March 2016).

[59] Sharko M, Jameson R, Ancker J et al. State-by State Variability in Adolescent Privacy Laws, *Pediatrics* 2022;149(6):e2021053458. 10.1542/peds.2021-053458 35531640

[60] CRC, General comment No, Doc UN. The right of the child to be heard. Volume 12. CRC/C/GC/12; 2009. (20 July 2009).

[61] CEDAW, General Recommendation No. 23 - Political and public life in Compilation of General Comments and General Recommendations adopted by human rights treaty bodies, Doc UN. HRI/GEN/1/Rev.6, (12 May 2003), pp. 260–271.

[62] CEDAW, General recommendation No. 21: Equality in marriage and family relations in International human rights instruments. compilation of General Comments and General Recommendations adopted by human rights treaty bodies (Vol. II), UN Doc. HRI/GEN/1/Rev.9(Vol.II), (27. May 2008), pp. 337–346.

[63] CRC, Concluding observations on the combined third and fourth periodic reports of India, Doc UN. CRC/C/IND/CO/3–4, (7 July 2014).

[64] CRC, Concluding observations on the combined fourth and fifth periodic reports of Jordan. UN Doc. CRC/C/JOR/CO/4–5, (8. July 2014).

[65] CRC, General comment No. 20, Doc UN. On the implementation of the rights of the child during adolescence. CRC/C/GC/20; 2016. (6 December 2016.

[66] CRC, General comment No. 4. (2003): Adolescent Health and Development in the Context of the Convention on the Rights of the Child, UN Doc. CRC/GC/2003/4, (1 July 2003).

[67] CRC, Concluding observations on the combined fifth and sixth periodic reports of Sri Lanka. UN, Doc. CRC/C/LKA/CO/5-6, (2 March 2018).

[68] CRC, General comment No. 4. (2003): Adolescent Health and Development in the Context of the Convention on the Rights of the Child, UN Doc. CRC/GC/2003/4, (1 July 2003).

[69] Report of the Special. Rapporteur on the right of everyone to the enjoyment of the highest attainable standard of physical and mental health, UN Doc. A/66/254, (4 April 2016).

[70] 2011, Communication No. 1608/2007: L.M.R. v Argentina, HRC, Doc UN. CCPR/C/101/D/1608/2007, (28 April 2011).

[71] UN., 1979, Convention on the Elimination of All Forms of Discrimination against Women.

[72] Alonso-Coello P, Schünemann HJ, Moberg J et al. GRADE Evidence to Decision (EtD) frameworks: a systematic and transparent approach to making well informed healthcare choices. 1: Introduction, *BMJ*, 2016;253;i2016. 10.1136/bmj.i2016 27353417

[73] del Carmen M, Joffe S (2005). Informed consent for Medical Treatment and Research: a review. Oncologist.

