# Supplementary material for: The impact of third-party authorization requirements on abortion-related outcomes: a synthesis of legal and health evidence
Source: BMC Public Health. 2023 Oct 23;23:2065. doi: 10.1186/s12889-023-16307-1 (PMC10591342; doi:10.1186/s12889-023-16307-1)
Supplement: Supplementary file 1 — Supplementary Material 1 [file 12889_2023_16307_MOESM1_ESM.docx]

### Supplementary Table 1. Evidence table: Impact of parental involvement requirements on abortion seekers

| **OUTCOME: DELAYED ABORTION** | | | | |
| --- | --- | --- | --- | --- |
| **Sub-outcome** | **Studies** | **Direction of evidence** | **What does this mean?** | **Overall conclusion** |
| -- | Ralph 2018 | ○ | Overall, parental notification laws are not associated with increasing gestational age among minors seeking abortions. | Evidence from two studies reporting on the association between parental notification requirement and delayed abortion are unclear. Variation in findings may be due to the study setting or inadequate sample size.  Requirements for parental notification are not observed to be associated with delayed abortion for all minors. However, minors who must travel outside their community to obtain abortion care experience significant delays in receiving care. |
|  |  | ▲ | Subpopulation: Among minors who must travel outside their community to obtain abortion care, parental notification laws are associated with a higher proportion of second trimester abortions compared with young adults (ages 18-21). |  |
|  | MacAfee 2015 | ○ | Parental notification requirement was not observed to be associated with an increase in gestational age among abortion seekers or in the number of second trimester abortions. |  |
| **OUTCOME: CONTINUATION OF PREGNANCY** | | | | |
| Unintended pregnancy rates | Medoff 2014c^3^ | ○ | Parental involvement laws are not associated with reductions in overall unintended pregnancy rates among minors and adults. | Overall, 14 studies across five sub-outcomes suggest that parental involvement laws for minors decrease abortion access and contribute to continuation of pregnancy.  The relationship between parental involvement laws and unintended pregnancy and birth rates suggests that overall, parental involvement laws increase adolescent birth rates but do not reduce unintended pregnancy or births.  When parental consent is associated with increased birth rates, there is a disproportionate impact on adolescents within specific populations (Black teens) and those engaged in cross-border travel. However, minors’ ability to travel to avoid parental consent laws is limited; the distance to a state without parental consent laws is irrelevant. |
| Abortion rates | Medoff 2010b | ⊽ | Parental involvement laws are associated with reduced overall abortion rates (minors and adults). |  |
|  | Medoff 2010c | ⊽ | Parental involvement laws are associated with reductions in overall abortion rates (minors and adults). |  |
|  | Medoff 2012b | ⊽ | Parental involvement laws are associated with reductions in overall abortion rate (minors and adults). |  |
|  | Ralph 2018 | ⊽ | Parental notification requirement is associated with a decrease in the number of abortions among minors (relative to numbers of abortions among young adults 18-21). |  |
|  | MacAfee 2015 | ⊽ | Parental notification requirement is associated with a decrease in the numbers of abortions among minors (attributed to a decrease in minors crossing into the state where the law applies). |  |
|  | Ramesh 2016^4^ | ○ | The impact of a parental notification requirement on the number of abortions among minors is unclear. |  |
|  | Joyce 2019 | ○ | Parental involvement laws adopted after 1995 were not observed to be associated with any decrease in adolescent abortion rates.  The effect of parental involvement laws on abortion rates is not impacted by distance to a setting without a parental consent law. |  |
|  | Tosh 2015 | ○ | Parental involvement laws are not associated with reduced adolescent abortion rates. |  |
| Unintended birth rates | Medoff 2016^5^ | ○ | Parental involvement laws are not associated with increasing unintended birth rates. |  |
| Birth rate | Medoff 2010a | **▲** | Parental involvement laws are associated with an increase in birth rates among unmarried adolescents. |  |
|  | Myers 2017 | **▲** | Parental involvement laws after the year 1992 are associated with an increase in birth rates among minors. Births rates increase with increasing distance to another setting (state) where parental involvement is not required. |  |
|  | Tosh 2015 | **▲** | Parental involvement laws are associated with increased adolescents birth rates. |  |
| Disproportionate impact | Myers 2017 | **▲** | Increased birth rates associated with parental consent laws are disproportionately experienced by Black teens.  Increased birth rates associated with parental consent laws are disproportionately experienced by individuals who must travel over 100 miles to avoid the parental consent law (travel to another region). |  |
|  | Medoff 2014a | ○ | Parental consent laws do not have a different effect on rates of abortion between white, Black or Hispanic women (15-44). |  |
| **OUTCOME: OPPORTUNITY COSTS** | | | | |
| Travel for abortion | Ralph, 2018 | ○ | Implementation of parental notification requirements do not impact on the number of minors and adult women engaged in cross-state travel to obtain abortion care.* | Evidence from four studies suggests that parental involvement laws are associated with increased opportunity costs for minors.  Parental involvement laws may lead to opportunity costs due to travel for abortion to states where parental consent or notification is not required, but do not prevent abortions from occurring.  Variation in findings may be due to specific differences in study settings. |
|  | MacAfee 2015 | ○ | Implementation of parental notification laws is associated with decreased travel into the state by abortion seekers living outside the state, but not associated with an increase in travel out of the state by those abortion seekers living within the state. |  |
|  | Fuentes 2019 | **▲** | Where parental involvement laws apply, minors are more likely to travel long distances (>100 miles) to access abortion. |  |
|  | Hung 2010^6^ | **▲** | Some minors will travel far to obtain unlawful abortion to avoid parental consent laws. |  |
| **OUTCOME: UNLAWFUL ABORTION** | | | | |
| -- | Hung 2010^6^ | **▲** | Some minors resort to unlawful abortion to avoid parental consent laws. | Evidence from one study suggests that parental consent laws may lead to unlawful abortion among minors. |
| **OUTCOME: ANTICIPATED EXPOSURE TO INTERPERSONAL VIOLENCE OR EXPLOITATION** | | | | |
| -- | Kavanagh 2012^6^ | **▲** | Minors are concerned that parental notification laws will expose them to physical and psychological violence during or after their pregnancy.  Minors are concerned that, if they continue their pregnancy, parental notification laws will expose their future children to violence. | Evidence from one study demonstrates that minors anticipate that involuntary disclosure of a pregnancy due to a requirement for parental notification may increase the risk for physical and psychological violence directed at them or their future children. |
| **OUTCOME: ANTICIPATED REPRODUCTIVE COERCION** | | | | |
| -- | Kavanagh 2012^6^ | **▲** | Minors expressed concern that parental notification laws will diminish their reproductive autonomy and lead to either a forced abortion or forced continuation of pregnancy. | Evidence from two studies indicate that minors anticipate that involuntary disclosure of a pregnancy due to a requirement of parental notification or consent, may increase the risk for reproductive coercion. |
|  | Hasselbacher 2014^6^ | **▲** | Minors' reasons for not wanting to disclose a pregnancy to a parent include a wish to preserve reproductive autonomy and avoid forced continuation of pregnancy. |  |
| **OUTCOME: ANTICIPATED FAMILY DISHARMONY** | | | | |
| -- | Kavanagh 2012^6^ | **▲** | Some minors anticipate that involuntarily disclosing their pregnancy due to parental notification requirements would result in a profound change in their relationship with their parent. | Evidence from two studies indicate that minors anticipate that involuntary disclosure of a pregnancy due to requirements for parental notification or consent may increase risk of family disharmony. |
|  | Hasselbacher 2014^6^ | **▲** | Some minors anticipate that involuntarily disclosing their pregnancy due to parental involvement laws would result in a profound change in their relationship with their parent. Reasons for not disclosing a pregnancy to a parent include a wish avoid anticipated family disharmony. |  |
| **OUTCOME: SYSTEMS COST** | | | | |
| Sexually transmitted infections | Colman 2013 | ○ | Parental involvement laws are not associated with increased rates of sexually transmitted infections, a proxy for sexual risk-taking behavior. | Overall evidence from six studies across six sub-outcomes suggest that parental involvement laws increase system costs.  Parental involvement laws have no impact on STI cases or pregnancy rates. Parental involvement laws may increase system costs related to pre-term birth and low birth weight, unwanted pregnancy rates and child homicide deaths. |
| Pregnancy rates | Medoff 2010d | ○ | Parental involvement laws are not associated with increased rates of adolescent pregnancies. |  |
| Unintended pregnancy rates | Medoff 2012a | ⊽ | Parental involvement laws are associated with an overall reduction in unintended pregnancy rates among minors and adults (ages 15-44). |  |
| Preterm birth and low birth weight | Wallace 2017 | **▲** | Parental involvement laws are associated with increased odds of preterm birth and low birth weight infants. |  |
| Postpartum depression | Medoff 2014b | ○ | Parental involvement laws are not associated with an increased occurrence of postpartum depression. |  |
| Homicide deaths among children <5 year | Sen 2012 | **▲** | Parental involvement laws are associated with an increased number of homicide deaths among children under age 5. |  |

▲ = the intervention leads to an increase in the outcome; ○ = the intervention leads to no change in the outcome; ⊽ = the intervention leads to a decrease in the outcome. Symbol does not indicate magnitude or certainty of effect.

*While the number of minors engaged in cross-border travel is not impacted by parental notification requirements, those that do travel are more likely to be in their second trimester when undergoing abortion.

^1^ Data are from a single institution, which affects generalizability.

^2^ Study was not powered to look at this outcome. Study population was restricted to pregnancies < 16 weeks, meaning that study results were biased towards showing no difference in gestational age or number of second trimester abortions.

^3^ Unintended pregnancy rates are from Kost et al (2013). *Unintended pregnancy rates at the state level: Estimates for 2002,2004, 2006 and 2008*. New York, NY: Guttmacher Institute.

^4^ Study was not powered to look at this outcome; no sample size calculation performed.

^5^ Unintended birth rates are from Sonfield, A., Kost, K., Gold, R. B., & Finer, L. B. (2011). The public costs of births resulting from unintended pregnancies: National and state-level estimates. Perspectives on Sexual and Reproductive Health, 43, 84–102.

6 Qualitative study design: tests of statistical significance not applicable.

### Supplementary Table 2. Evidence table: Impact of judicial bypass on abortion seekers

| **Studies** | **Direction of evidence** | **What does this mean?** | **Overall conclusion** |
| --- | --- | --- | --- |
| **OUTCOME: DELAYED ABORTION** | | | |
| Coleman-Minahan 2019^1^ | ▲ | Minors using judicial bypass experience delays in accessing abortion (median 17 days; range: 2 days-8 weeks). | Evidence from two studies suggests that judicial bypass may be associated with delayed abortion. |
| Ralph 2021 | ▲ | Minors using judicial bypass experience delays in accessing abortion (mean 6.4 days between contact with court and court hearing, range 0-27 days). |  |
| **OUTCOME: OPPORTUNITY COSTS** | | | |
| Coleman-Minahan 2019^1^ | ▲ | Minors using judicial bypass when independent consent is not permitted experience logistical burdens (e.g., travel coordination & costs, mandatory appointments with missed time at school, work & home, care coordination with clinics) when having to travel to and from the courthouse. | Evidence from four studies supports that judicial bypass may be associated with opportunity costs  Some minors need a confidential pathway to obtain abortion care. These minors report meaningful logistical burdens and opportunity costs in obtaining an abortion by judicial bypass. |
| Kavanagh 2012^1^ | ▲ | Minors report that the need for judicial bypass would complicate the process significantly (e.g., difficulties finding free or affordable legal services, making an appointment with a judge, transportation, missed time from school). |  |
| Maffi & Affes 2019^2^ | ▲ | Minors using judicial bypass sometimes experience multiple opportunity costs, including uncertainty and delays, when the responsibility in terms of the final decision to allow the abortion is passed between judge and healthcare providers. |  |
| Ralph 2021 | ▲ | Delays associated with judicial bypass push some minors (6/128) over local thresholds for medical abortion.  Judicial bypass is associated with substantial travel time especially for those residing far from court house (mean distance travelled=39 kilometers). |  |
| **OUTCOME: ANTICIPATED EXPOSURE TO INTERPERSONAL VIOLENCE OR EXPLOITATION** | | | |
| Coleman-Minahan 2019^1^ | ⊽ | Minors use judicial bypass when independent consent is not available in order to avoid anticipated violence. | Evidence from four studies indicates that minors value and need a pathway to obtain confidential abortions. Minors request judicial bypass when they anticipate violence if a pregnancy is disclosed. Judicial bypass may decrease anticipated violence by creating a pathway where minors can obtain confidential abortions. |
| Coleman-Minahan 2020^1^ | v | Minors use judicial bypass when independent consent is not available in order to avoid anticipated violence. |  |
| Friedman 2015 | ⊽ | Minors use judicial bypass when independent consent is not available in order to avoid anticipated violence. |  |
| Ralph 2021 | ⊽ | Minors report (16/128) using judicial bypass out of concern that a parent/s would otherwise expose them to physical or emotional abuse. |  |
| **OUTCOME: ANTICIPATED REPRODUCTIVE COERCION** | | | |
| Coleman-Minahan 2020^1^ | ⊽ | Minors' reasons for not wanting to disclose a pregnancy to a parent and their use of judicial bypass include a wish to preserve reproductive autonomy and avoid forced continuation of pregnancy. | Evidence from three studies indicates that minors value and need a pathway to obtain confidential abortions.  Minors request judicial bypass when they anticipate reproductive coercion if a pregnancy is disclosed.  Judicial bypass may decrease the risk of reproductive coercion by creating a path where minors can have confidential abortions. |
| Friedman 2015 | ⊽ | Minors use judicial bypass when independent consent is not available in order to avoid anticipated reproductive coercion. |  |
| Ralph 2021 | ⊽ | Minors report (64/128) using judicial bypass out of concern that a parent/s would otherwise force them to carry their pregnancy to term. |  |
| **OUTCOME: ANTICIPATED FAMILY DISHARMONY** | | | |
| Coleman-Minahan 2020^1^ | ⊽ | Some minors anticipate that involuntarily disclosing their pregnancy would result in a profound change in their relationship with their parent and therefore they use judicial bypass to access abortion. | Evidence from three studies indicates that minors value and need a pathway to obtain confidential abortions.  Minors request judicial bypass when they anticipate family disharmony if a pregnancy is disclosed.  Judicial bypass may decrease family disharmony by creating a path where minors can have confidential abortions. |
| Friedman 2015 | ⊽ | Minors use judicial bypass when independent consent is not available in order to avoid being kicked out of home, and creating a poor relationship with parents' family religious values. |  |
| Ralph 2021 | ⊽ | Minors report (35/128) using judicial bypass out of concern that the pregnancy would otherwise ruin their relationship with family. 52/128 report using judicial bypass to end their pregnancy out of concern that they would otherwise be cut off financially or kicked out of home by parent/s. |  |

▲ = the intervention leads to an increase in the outcome; ○ = the intervention leads to no change in the outcome; ⊽ = the intervention leads to a decrease in the outcome. Symbol does not indicate magnitude or certainty of effect.

^1^ Qualitative study design: tests of statistical significance not applicable.

^2^ Concerns about adequacy exist – data underlying the finding is not sufficiently rich, data come from a small number of studies and few participants.

### Supplementary Table 3. Evidence table: Impact of judicial bypass vs. parental consent on abortion seekers

| **Sub-outcome** | **Studies** | **Direction of evidence** | **What does this mean?** | **Overall conclusion** |
| --- | --- | --- | --- | --- |
| **OUTCOME: DELAYED ABORTION** | | | | |
| -- | Altindag 2017^1^ | ⊽ | Minors using judicial bypass compared with parental consent experience shorter delays in obtaining abortion. | Evidence from three studies examining the difference between judicial bypass and parental consent on delayed abortion is unclear. Differences in estimates may be due to significant variation in the bypass process across settings.  When judicial bypass is associated with greater delays compared with parental consent, minors using judicial bypass are more likely to pass gestational thresholds for medical abortion per local guidance.  Evidence from two studies suggest that specific populations of minors are more likely to use judicial bypass than parental consent to obtain an abortion, and thus may be disproportionately impacted by the effects of judicial bypass. |
|  | Janiak 2019^1^ | ▲ | Minors using judicial bypass compared with parental consent experience greater delays in obtaining abortion. |  |
|  | Joyce 2010^1^ | ⊽ | Minors using judicial bypass compared with parental consent undergo abortions at earlier gestational ages, and are less likely to have a second trimester abortion. |  |
| Passing gestational thresholds | Janiak 2019 | ▲ | Minors using judicial bypass compared with parental consent, are more likely to pass gestational thresholds that render them ineligible for medical abortion (per local guidance). |  |
| Disproportionate impact | Janiak 2019 | ▲ | Minors requesting abortion by judicial bypass compared with parental consent vary significantly. Minors obtaining abortion under judicial bypass as compared with parental consent are significantly more likely to be racial or ethnic minorities and of low socioeconomic status. |  |
|  | Joyce 2010 | ▲ | Minors seeking an abortion by judicial bypass as compared with parental consent, are more likely to be Hispanic, be under 15 years of age, and be an out of state resident. |  |

▲ = the intervention leads to an increase in the outcome; ○ = the intervention leads to no change in the outcome; ⊽ = the intervention leads to a decrease in the outcome. Symbol does not indicate magnitude or certainty of effect.

^1^Symbol indicates the directionality of the outcome - delayed abortion, as there are no sub-outcomes.

### Supplementary Table 4: Parental consent vs. notification: impact on abortion seekers (summary)

| **Outcome** | **Overall conclusion of evidence** | **Applicable Human Rights Standards** | **Conclusion evidence + Human Rights** |
| --- | --- | --- | --- |
| Delayed abortion | Evidence from one study examining the difference between parental consent statute vs. notification requirement suggests there is no difference on delayed abortion. | TPAs engage states’ obligations to respect, protect and fulfil the rights to life and health (by taking steps to reduce maternal mortality and morbidity), and to equality and non-discrimination (because of disproportionate impact on vulnerable groups). | As delay is associated with increased maternal mortality and morbidity, and as delay associated with parental consent laws has disproportionate impact on specific populations, and as a parental notification requirement rather than parental consent requirement does not change associated abortion delay, these laws are associated with reduced enjoyment of the right to health, the right to life, and the right to equality and non-discrimination. |
| Continuation of pregnancy | Evidence from one study suggests that parental consent laws in comparison with parental notification, decrease minors’ abortion rates to a greater extent and contribute to continuation of pregnancy. | TPAs engage states’ obligations to protect, respect and fulfil the right to health, the right to decide on the number and spacing of children, the right to privacy, and the right to equality and non-discrimination. | As delay is associated with increased maternal mortality and morbidity, and as delay associated with parental consent laws has disproportionate impact on specific populations, and as a parental notification requirement rather than parental consent requirement does not change associated abortion delay, and as written parental consent requirements may be associated with lower abortion rate and higher birth rates, these laws are associated with reduced enjoyment of the right to health, the right to life, and the right to equality and non-discrimination. |
| Opportunity costs | No studies identified | -- |  |
| Unlawful abortion | No studies identified | -- |  |
| Self-managed abortion | No studies identified | -- |  |
| Anticipated exposure to violence or exploitation | No studies identified | -- |  |
| Anticipated reproductive coercion | No studies identified | -- |  |
| Anticipated family disharmony | No studies identified | -- |  |
| System costs | No studies identified | -- |  |

### Supplementary Table 5. Evidence table: Impact of parental consent vs. notification on abortion seekers

| **Sub-outcome** | **Studies** | **Direction of evidence** | **What does this mean?** | **Overall conclusion** |
| --- | --- | --- | --- | --- |
| **OUTCOME: DELAYED ABORTION** | | | | |
| -- | Joyce 2010 | ○ | Minors using parental consent statues vs. parental notification to obtain abortion do not have different rates of second trimester abortion. | Evidence from one study examining the difference between parental consent statute vs. notification requirements suggests there is no difference on delayed abortion. |
| **OUTCOME: CONTINUATION OF PREGNANCY** | | | | |
| Abortion rate | Chevrette 2015 | ⊽ | Settings with mandatory parental consent laws as compared with parental notification or minor’s own ability to consent, may have lower abortion rates. | Evidence from one study suggests that parental consent laws in comparison with parental notification, decrease minors’ abortion rates to a greater extent and may contribute to continuation of pregnancy. |
| Adolescent birth rate | Chevrette 2015^1^ | ○ | States with mandatory parental consent laws as compared with parental notification or minor’s own ability to consent, have no difference in adolescent birth rates. |  |

▲ = the intervention (parental consent) leads to an increase in the outcome; ○ = the intervention leads to no change in the outcome; ⊽ = the intervention leads to a decrease in the outcome. Symbol does not indicate magnitude or certainty of effect.

^1^ Study was not powered to look at this sub-outcome (p.07) and model did not adjust for all known confounders.

### Supplementary Table 6: Spousal consent: impact on abortion seekers (summary)

| **Outcome** | **Overall conclusion of evidence** | **Applicable Human Rights Standards** | **Conclusion evidence + Human Rights** |
| --- | --- | --- | --- |
| Delayed abortion | No studies identified. | TPAs engage states’ obligations to respect, protect and fulfil the rights to life and health (by taking steps to reduce maternal mortality and morbidity), and to equality and non-discrimination (because of disproportionate impact on vulnerable groups). | If spousal consent requirements result in delayed abortion this may impact negatively on the rights to life, health, and equality and non-discrimination. |
| Continuation of pregnancy | No studies identified. | TPAs engage states’ obligations to protect, respect and fulfil the right to health, the right to decide on the number and spacing of children, the right to privacy, and the right to equality and non-discrimination. | If spousal consent requirements result in unwanted continuation of pregnancy this may impact negatively on the rights to life, health, privacy, equality and non-discrimination, and the right to decide the number and spacing of children. |
| Opportunity costs | No studies identified. | TPAs engage states’ obligations to protect, respect and fulfil the right to health by ensuring abortion regulation is evidence-based and proportionate, that where it is lawful abortion is safe and accessible. | If spousal consent requirements result in opportunity costs this may impact negatively on the right to health and to equality and non-discrimination. |
| Unlawful abortion | Evidence from one study suggest that some women will resort to unlawful abortion in order to avoid spousal consent. | TPAs engage states’ obligations to respect, protect and fulfil the rights to life and health (by taking steps to reduce maternal mortality and morbidity, and by protecting people from the physical and mental health risks associated with unsafe abortions). | As spousal consent requirements apply only to married women and may lead to unlawful abortion, and as unlawful abortion is associated with increased maternal mortality and morbidity, making abortion less safe and accessible, giving rise to physical and mental health risks, these requirements are associated with reduced enjoyment of the right to life, health, and equality and non-discrimination. |
| Self-managed abortion (SMA) | No studies identified. | TPAs engage states’ obligations to respect, protect and fulfil the rights to life and health (by taking steps to reduce maternal mortality and morbidity, and by protecting people from the physical and mental health risks associated with unsafe abortions). | If spousal consent requirements result in recourse to SMA and if such SMA is unsafe, spousal consent requirements impact negatively on abortion seekers’ rights to life, health, and equality and non-discrimination. |
| Anticipated exposure to violence or exploitation | No studies identified. | TPAs engage states’ obligations to protect abortion seekers, the right to privacy, life, health, and equality and non-discrimination. | If spousal consent requirements result in exposure to violence or exploitation, these requirements impact negatively on abortion seekers’ rights to life, health, and equality and non-discrimination. |
| Anticipated reproductive coercion | No studies identified. | TPAs engage states’ obligations to protect the right to health, right to security of person, right of persons with disabilities to retain fertility on an equal basis with others, right to be free from torture, and cruel, inhuman or degrading treatment, right to exercise legal capacity, right to decide on the number and spacing of children, right to equality and non-discrimination, right to privacy, and women’s right to legal capacity on an equal basis with men. | If spousal consent requirements result in reproductive coercion, these requirements impact negatively on abortion seekers’ rights including rights to life, health, and equality and non-discrimination. |
| Anticipated family disharmony | No studies identified. | TPAs engage states’ obligations to protect the right to health, right to security of person, right of persons with disabilities to retain fertility on an equal basis with others, right to be free from torture, and cruel, inhuman or degrading treatment, right to exercise legal capacity, right to decide on the number and spacing of children, right to equality and non-discrimination, right to privacy, and women’s right to legal capacity on an equal basis with men. | If spousal consent requirements result in family disharmony, these requirements may impact negatively on abortion seekers’ rights including rights to life, health, security of person, and equality and non-discrimination. |
| System costs | No studies identified. | TPAs engage states’ obligations to protect, respect and fulfil the right to health (by ensuring that where it is lawful abortion is safe and accessible, and that regulation of abortion is evidence-based and proportionate). | If spousal consent requirements increase system costs, they may have negative impacts on the right to health. |

*Supplementary Table 7: Evidence table: Impact of spousal consent on abortion seekers*

| **Studies** | **Direction of evidence** | **What does this mean?** | **Overall conclusion** |
| --- | --- | --- | --- |
| **OUTCOME: UNLAWFUL ABORTION** | | | |
| MacFarlane 2016^1, 2^ | **▲** | When spousal consent laws are present, women experience reproductive coercion, and some will resort to unlawful abortion. | Evidence from one study suggest that some women will resort to unlawful abortion in order to avoid spousal consent. |

▲ = the intervention (spousal consent) leads to an increase in the outcome; ○ = the intervention leads to no change in the outcome; ⊽ = the intervention leads to a decrease in the outcome. Symbol does not indicate magnitude or certainty of effect.

^1^ Qualitative study design: tests of statistical significance not applicable.

^2^ Concerns about adequacy exist – data underlying the finding is not sufficiently rich, data come from a small number of studies and few participants.
